# Are face transplant candidates choosing autonomously? A preliminary method to evaluate autonomous choosing in psychosocial and bioethical assessments

**DOI:** 10.3389/frtra.2024.1346667

**Published:** 2024-02-27

**Authors:** Anneke Farías-Yapur

**Affiliations:** Interdisciplinary Center for Bioethics, Universidad Panamericana, Mexico City, Mexico

**Keywords:** informed consent, deferential vulnerability, self-worth, vascularized composite allotransplantation, autonomy, bioethical assessment

## Abstract

This report proposes a framework for evaluating the validity of informed consent and autonomy in face transplant candidates, taking into account the risk of depression and non-compliance. Traditional factors like decisional capacity, disclosure, comprehension, voluntariness, and agreement are insufficient for assessing valid informed consent in individuals whose self-worth relies on public perception, potentially leading to self-harm if societal worth is undermined. Reliance on self-esteem, rather than inherent personal value, poses risks of depression, poor treatment adherence, and deferential vulnerability. We suggest a qualitative analysis of self-worth, self-esteem, self-trust, and self-respect to better assess the autonomy of face transplant candidates in their decision-making process.

## Introduction

Transplant surgeries carry life-long risks, including graft rejection and therapy-related complications (1). While procedures like heart, lung, and liver transplants are performed to save lives, face transplantation is distinct. It aims to enhance quality of life by addressing esthetics and functionality. However, facial tissue's high antigenicity increases the risk of rejection, whether acute or chronic (2, 3). Chronic rejection may lead to neurofibromatosis and graft loss, necessitating retransplants (4). To prevent this, chronic immunosuppression is required, albeit with a higher risk of malignancy (5). In addition, the financial burden remains substantial, particularly for patients without healthcare coverage, as seen in the case of Mexican patients.

Although many patients may seek transplantation for functional purposes, “the vast majority of the psycho-emotional suffering experienced by a disfigured individual [is] a direct result of societal perception,” therefore shoring patients to accept a facial transplant as a “desperate means of ridding themselves of disfigurement” and the subsequent discrimination. The first face transplant patient “highlights the importance of social responses to facial disfigurement and the extraordinary pressures on patients to conform, whatever the cost” (6). Consenting to a life-threatening intervention to conform to social standards of beauty can be considered pertaining to deferential vulnerabilities: powerful social and cultural pressures that prompt patients to “accede to the perceived desires of certain others notwithstanding an inner reticence to do so” (7). Such vulnerability—among others—compromise consent (7).

Practitioners must assess various factors to determine the validity of patients’ informed consent, including competence, disclosure, comprehension, voluntariness, and agreement (8, 9). While psychological screenings are commonly used to evaluate transplant candidates’ capacity for informed consent, they mainly focus on cognitive vulnerabilities that may affect understanding. However, the psychological aspects of voluntariness and agreement are often overlooked due to their subjective nature, making them challenging to measure and susceptible to deferential vulnerabilities. To date, clear standards for addressing these issues have not been established (10).

Considering that “the vast majority of the psycho-emotional suffering experienced by a disfigured individual [is] a direct result of societal perception” (11) and that evaluating voluntariness and agreement are hardly measurable, we wonder whether, by giving consent to receive a life-threatening intervention and accepting higher risks than other candidates, face transplant candidates could be showing a sign of a deferential vulnerability. This not only highlights an important bioethical problem regarding the autonomy with which some patients could be agreeing to such a procedure, but also suggests that for some, the graft—that has to be maintained with life-long efforts, risks, and financial costs—is a means to attain an objective which cannot be guaranteed.

To our knowledge, current psychosocial evaluations of patients’ suitability for face transplantation do not specifically focus on assessing autonomy in relation to deferential vulnerabilities. Consequently, we aim to propose a theoretical framework that can guide the evaluation of informed consent and autonomy in face transplant candidates while considering deferential vulnerabilities. Our approach draws upon a Kantian interpretation of Honneth's Recognition Theory (12), which can help us understand a patient's susceptibility to depression, a significant post-surgery risk factor.

### Preconditions for autonomous choosing

Autonomy, a fundamental principle in bioethics (13), signifies self-governance and links to personal causation (14). It forms the bedrock of informed consent's validity (15–19), a pivotal concept in bioethics and medical practice (20). Autonomy, deeply rooted in Western moral philosophy, relies on practical reason—concerned with what one should do rather than objective truth [(21, 22); *Critique of Pure Reason*, A800–801/B828–829]. Within practical reason, there exist psychological underpinnings for autonomous decision-making (23). Honneth's Theory of Recognition posits three foundational self-relationships preceding autonomy: self-trust, arising from love, allows us to trust our emotions [(12), p. 95]; self-respect, the ability to view oneself as morally responsible (p. 118); and self-esteem, recognizing one's achievements and abilities as valuable to society (p. 130).

### A Kantian reading of the self-relationships

Although these definitions highlight relevant social values, we believe that it is important to also think about the self-relationships described through the light of a Kantian vision to further highlight a non-movable value—human dignity. The notion of value in general can only exist in the context of the comparison of an object in regards to a criterion which grants worth. In that sense, criteria by which self-worth adjusts are, at least in Western culture, two: on the one hand—and this is referred to as oppressive socialization (23–25)—adjustment to social standards of beauty, wealth, intelligence, etc. Another way to call the resulting worth is “contingent self-esteem” (26), which has also been called “contingent self-worth” (27). Deci and Ryan (26), affirm that “Contingent self-esteem refers to feelings about oneself that result from—indeed, are dependent on—matching some standard of excellence or living up to some interpersonal or intrapsychic expectations” (p. 32). On the other hand, the criterion by which self-worth adjusts is—as defended by Kant [(27) p. 428-429]—dignity as an axiom which allows no degrees but only an absolute value, no matter how many ways a person fails to achieve social standards. For discussions regarding the groundings of dignity, see Formosa (23).

Self-relationships are intimately related to self-worth. To explain how, we chose to define the aforementioned self-relationships through dignity and the notion of respect. In a Kantian sense, respect can be thought of as impeding oneself to treat another merely as a means rather than an end [cf. (29), p. 65]. It can be further thought of as impeding oneself to abuse someone's vulnerable position in which he or she could be treated as not deserving dignity —due to reasons such as those conveyed by oppressive socialization [cf. (24)]. Self-respect could therefore be seen as the ability to treat oneself with dignity —as a person with an absolute value that cannot be achieved and therefore cannot be lost regardless of failures and shortcomings [cf. (30), p. 883-7]. Self-respect allows self-worth to rest solely upon dignity as an axiom, and impedes it from decaying if failing to achieve social recognition [(31), Section II, para. 428).

Following this line, trust can be thought of as the resulting expectation that comes from respecting dignity regardless of imperfections, and so avoiding worth decaying when one or another is faced with shortcomings. Therefore, self-trust is the expectation that one will preserve worth as an absolute value, regardless of socially rejected limitations. That is, as the expectation of one's dignity to be defended and treated by oneself with respect, regardless of failing to achieve social standards—such as those of beauty—that oppressively subjugate people's self-recognition of dignity and worth.

Finally, following the sociometer theory (32) self-esteem “is a psychological gauge of the degree to which people perceive that they are relationally valued and socially accepted by other people”. It can be seen as an attitude toward oneself as if one were adopting the perspectives and judgments of others, making it dependent on what society deems praiseworthy or condemnable [(33), p. 90]. Unlike the previously discussed self-relationships, self-esteem naturally corresponds to one's belief in their conformity to societal standards (34). Consequently, self-esteem varies with personal achievements and the level of social recognition received (27). If the criterion for self-worth is based on one's perception of meeting societal expectations to gain social approval (27), then self-worth would be equivalent to self-esteem (26). However, since self-esteem fluctuates and lacks an immutable axiom for the stability of self-worth, equating self-esteem with self-worth implies that an individual sees themselves as devoid of inherent dignity. This is why some people may maintain high self-esteem while feeling worthless unless they continuously demonstrate their worthiness and dignity through achievements and social approval. This dynamic could help explain why blows to self-esteem, followed by subsequent shame, are potent risk factors for suicide in certain individuals (35). We contend that tying self-worth to self-esteem leads to heteronomous rather than autonomous decision-making [(23), p. 204].

Grounding self-worth in self-esteem presents additional issues beyond autonomous decision-making, such as in face transplantation cases. While not all individuals resort to suicide when facing reduced self-esteem and shame, it is well-documented that damaged self-esteem often leads to depression (36, 37). Depression is closely linked to poor treatment adherence (38), potentially putting face transplant patients at risk of death. Consequently, psychosocial evaluations of face transplant candidates routinely include assessments for depression (39–42).

### Assessing self-relationships and deferential vulnerability in face transplantation

To our knowledge, standardized tests evaluating the self-relationships as defined previously have not yet been developed. However, while performing bioethical assessments of Mexico's two first face transplant candidates, we conducted clinical interviews to—among others—understand the patients’ self-relationships and main motivations to seek transplantation.

If the patient seeks a face transplant to increase self-esteem (via diminished social rejection), and so to conceal his or her notion of low self-worth, deferential vulnerability to accept the risks of receiving a face transplant is likely to be present. If the patient's main motivation to seek the transplant is to achieve real or imagined family expectations that would not be attained, or to increase his or her self-worth because it is dependent on public perception, then failing to achieve this through constant social rejection may result in significant grief, since face transplantation and its accompanying burdens are non-reversible. This could increase the likelihood of having depression, which—in addition to lacking material and social support [see (43)]—would affect adherence [(44); cf (45)].

To assess the aforementioned, we performed qualitative analysis of clinical interviews. Sessions evaluating microsystemic and individual factors [see (43)] relevant to autonomous choosing, aimed at four main objectives:

(1) To evaluate the patients’ self-relationships to know whether their self-worth was dependent on the public perception of the self. For this, the evaluated topics were: degree of self-respect by analyzing self-esteem as a threat to self-worth; deterioration of self-esteem and suicide risk (resilience and adherence to treatment); degree of self-respect by analyzing the independence of self-worth from self-esteem; self-esteem with protected self-worth. Along all these dimensions, shame—as a powerful feeling and attitude consequence to devaluing self-worth—was carefully monitored, and understood as a relevant indicator of potential risk factors for depression, as well as suicidality (46, 47), and probably as a potent incentive to receive the face transplantation as a reparative means [cf (11)].
(2) To evaluate both the patients’ and their family's main motivations for the patients receiving the transplant. We assessed whether the main motivations of either part aimed at concealing notions of low self-worth; and therefore, comprised powerful social and cultural pressures that prompt patients to accede to the perceived desires of certain others to receive the face transplant, notwithstanding an inner reticence to do so [cf (7, 11)].
(3) To know what the patient would do if his objectives (such as social acceptance) weren’t going to be achieved the way he hoped—as was the case of the first face transplant patient (6). The mental exercise of failing to achieve the main motivations for which they seek transplantation may help them not to overestimate benefits and overlook risks, as is often the case with desperate people [cf (7)]. When inspecting this topic in relation to the previous ones, it is possible that hidden pressures which were promoting patients to accept such a life-long high-risk intervention are uncovered and reflexively analyzed, further helping them carefully assess the risk–benefit ratio of receiving a face transplant. In addition, this mental exercise may help them analyze whether they can achieve their main motivations through strategies other than face transplant. Finally, it may help them notice—should they still want transplantation—other desirable outcomes not related to their main motivation, that could serve as indicating success even if the main motivations were not to be achieved, therefore potentially protecting them from grief and depression-non-adherence.
(4) To know the material and social resources, as well as their probable stability through time, to support the patient's recovery and subsequent treatment [see (43)]. From a social-ecological understanding of resilience, treatment adherence and recovery are not solely dependent on individual factors and personal will, but it rather depends on the material and social support the context provides, as well as the meaningfulness the patients assign to those resources as supportive to recovery [cf. (43, 48)].

Since the differentiation of self-esteem from self-worth is not always evident, it is recommended that sessions with the patient be recorded, and that multiple sessions evaluate the self-relationships. A verbatim transcription of the interviews helps identify fragments discussing the self-relationships as well as their interplay in the context of a particular situation—such as social rejection, achieving or failing to achieve the main motivations, and others. Based on the analysis of the topics, an initial assessment can be provided—as well as the resources or the obstacles to the development of self-respect and self-worth independently from self-esteem.

## Discussion

The present report seeks to expand on the factors that should be evaluated before granting autonomous choosing in face transplant candidates. Following Theodorakopoulou et al. (11) and Kipnis (7), we hoped to give helpful lines of thought to analyze whether social and cultural pressures prompt face transplant patients to accept higher risks than any other transplant patients, notwithstanding their inner reticence to do so.

Drawing from Kantian interpretations of Honneth's practical self-relationships, we argue that assessing self-respect in relation to self-worth and dignity as well as the independence of self-worth from self-esteem are crucial psychological considerations when evaluating the autonomous decision-making of face transplant candidates. Furthermore, examining these psychological factors may shed light on susceptibility to depression, a significant risk factor for treatment adherence. Our analysis suggests that anchoring self-worth in self-esteem, rather than inherent personal value, can make individuals vulnerable to deferential influences, potentially leading to non-autonomous choices and depression, with subsequent implications for treatment adherence. While we propose topics for clinical interview analysis, the development of reliable scales specifically assessing these self-relationships is essential for comprehensive evaluation.

We believe it would be unfair to assert that an autonomous face transplant candidate is one who has no influence from the desire to eliminate social discrimination, or that wanting to get rid of social rejection amounts to deferential vulnerability. That is why, by ruling out the achievement of increasing their social value, we evaluate whether the other transplant outcomes compete the same way with the risks and life-long sacrifices that come with the transplant. It is crucial not to undervalue the risk for neurofibromatosis and the need for a retransplant (4) the cancer risk associated with the drugs enabling the transplant (6), the economic burden (1), the continued stigma faced by real individuals (6), and their genuine suffering. We do not want to underestimate the pain and suffering borne by disfigurement, which must be acknowledged and ethically managed. However, we believe it is their pain and suffering, largely caused by social rejection, that make this vulnerable population prone to desperately choose shame-reparative strategies while overlooking the significant risks of a procedure that aims to provide them with a more “normalized” appearance [cf (6, 11)].

Critics of a reluctant stance to prescribe face transplants might argue that it is the careful selection of patients that best predict “success” cases, and that “failed” cases might be simply “not ideal patients”. However, we believe this approximation could miss reflecting on the bioethical dilemma of whether one should perform face transplants in the first place (6). Such an attitude might as well dismiss thinking about the patients’ subjective grounds by which face transplant procedures should be considered as “successes” or “failures.” Literature has not made a deep exploration of patients’ psychological development and experience after surgery, since literature describing patient follow up mostly deals with the mechanics of their physical recovery (6). An example thereof can be found in the description of the physical recovery of a French patient, who after 7 years of chronic graft rejection and neurofibromatosis, required a retransplant, for which he had to endure 6 weeks without a graft, while also having panic attacks and psychotic episodes (see Lantieri et al. 2020).

In that sense, there is scarcity of knowledge of patients’ experience of life after a face transplant both in the short and long-term, therefore lacking the very foundations that should inform not only understanding of efficacy, success, failure of face transplants but also future candidates’ informed consent.

Since consent based on knowledge and understanding of other patients’ life after the transplant is not available yet, we believe all programs that provide face transplants should come with efforts to foster candidates’ self-acceptance, self-worth, and self-esteem *before* they consent to a potentially life-enhancing non-life-saving procedure—and patients who already underwent such procedure could be greatly benefited from it too. Changing Faces in the United Kingdom (11) is an organization that helps people with deformities accept themselves and achieve *a fulfilled quality of life* (https://www.changingfaces.org.uk/).

Combining efforts both to assess autonomous choosing of face transplants as well as providing interventions such as the one provided by Changing Faces would be highly beneficial—not only to better ensure that patients who consent to receive a face transplant consent with autonomy but also as a life-enhancing intervention without the subsequent risks implied with the life-long journey to preserve the facial graft.

## Data Availability

The original contributions presented in the study are included in the article/Supplementary Material, further inquiries can be directed to the corresponding author.
