# Corrigendum: Are face transplant candidates choosing autonomously? A preliminary method to evaluate autonomous choosing in psychosocial and bioethical assessments

**DOI:** 10.3389/frtra.2024.1433414

**Published:** 2024-06-14

**Authors:** Anneke Farías-Yapur

**Affiliations:** Interdisciplinary Center for Bioethics, Universidad Panamericana, Mexico City, Mexico

**Keywords:** informed consent, deferential vulnerability, self-worth, vascularized composite allotransplantation, autonomy, bioethical assessment

A Corrigendum on Are face transplant candidates choosing autonomously? A preliminary method to evaluate autonomous choosing in psychosocial and bioethical assessments By Farías-Yapur A (2024). Front. Transplant. 3:1346667. doi: 10.3389/frtra.2024.1346667

**Incorrect Funding**

In the published article, there was an error in the Funding statement. The statement did not acknowledge the funding that made it possible to publish the paper. The original text stated: “The author declares that no financial support was received for the research, authorship, and/or publication of this article.” The correct Funding statement appears below.

